# Lipopolysaccharide Alters the m6A Epitranscriptomic Tagging of RNAs in Cardiac Tissue

**DOI:** 10.3389/fmolb.2021.670160

**Published:** 2021-07-28

**Authors:** Ye-Chen Han, Hong-Zhi Xie, Bo Lu, Ruo-Lan Xiang, Hai-Peng Zhang, Jing-Yi Li, Shu-Yang Zhang

**Affiliations:** ^1^ Department of Cardiology, Peking Union Medical College Hospital, Chinese Academy of Medical Sciences and Peking Union Medical College, Beijing, China; ^2^ Department of Physiology and Pathophysiology, Peking University School of Basic Medical Sciences, Beijing, China; ^3^ Peking University Fifth School of Clinical Medicine (Beijing Hospital), Beijing, China

**Keywords:** N6-methyladenosine, sepsis, myocardial dysfunction, epitranscriptomics, lncRNA

## Abstract

N6-methyladenosine (m^6^A) modification plays important roles in the pathology of a variety of diseases. However, the roles of m^6^A modification in sepsis-induced myocardial dysfunction are not well defined. Rats were divided into control and lipopolysaccharide (LPS)-induced sepsis group. Global m^6^A levels of left ventricle tissue were measured by LC-MS/MS, and transcriptome-wide m^6^A modifications were profiled using epitranscriptomic microarrays (mRNAs and lncRNAs). Bioinformatics analysis was conducted to understand the functional implications of m^6^A modifications during sepsis. Methylated lncRNAs and mRNAs were measured by m^6^A single-base site qPCR. The global m^6^A levels in left ventricle tissue were significantly decreased in the LPS group. While 27 transcripts (23 mRNAs and four lncRNAs) were hypermethylated, 46 transcripts (39 mRNAs and 7 lncRNAs) were hypomethylated in the LPS group. The mRNA expression of writers and readers was significantly decreased in the LPS group. The m^6^A modification of Clec1b, Stk38l and Tnfrsf26 was associated with platelet activation and apoptotic pathways. Moreover, the decrease in m^6^A modification of lncRNA XR_346,771 may be related to cation import in cardiac tissue. Our data provide novel information regarding changes to m^6^A modifications in cardiac tissue during sepsis, and m^6^A modifications might be promising therapeutic targets.

## Introduction

Sepsis is a life-threatening condition involving organ dysfunction that is caused by a dysregulated host response to infection (Cohen et al., 2015), It has been reported that approximately 50% of patients with septic shock are diagnosed with septic cardiomyopathy, which causes myocardial dysfunction, and these patients tend to have poor prognoses (Zaky et al., 2014). Myocardial dysfunction is an important factor that contributes to the high mortality rate of sepsis. Therefore, it is necessary to elucidate the pathogenesis of myocardial dysfunction in order to develop new treatment strategies to reduce the mortality rate of sepsis.

To date, a series of reversible posttranscriptional modifications located in RNAs have gained increasing attention. More than 140 chemical modifications have been discovered in RNAs. The modifications range from simple methylation or isomerisation, such as N6-methyladenosine (m^6^A), 5-methylcytosine (m5C), N1-methyladenosine (m^1^A), pseudouridine (Ψ), 5-methyluridine (m^5^U),1-methylguanosine, and 7-methylguanosine (m^1^G and m^7^G, respectively) and inosine (I), to complex multistep chemical modifications, such as N^6^-threonylcarbamoyladenosine and 5-methoxycarbonyl-methyl-2-thiouridine (mcm5s^2^U) (Pan, 2018). Among them, the m^6^A is one of the most abundant and influential modifications in eukaryotes. M^6^A modification is involved in regulating a variety of posttranscriptional events, including pre-miRNA processing and RNA stability, translation and alternative splicing (Chokkalla et al., 2019). M^6^A modifications occur via the m^6^A methyltransferases called “writers”; they are removed by the demethylases called “erasers” and are recognized by m6A-binding proteins called “readers”. The orchestrated interplay among writers, erasers, and readers drives the dynamics and outcomes of the m^6^A modification of RNAs. An increasing number of studies have reported that m^6^A modification plays important roles in biological and pathophysiological processes, such as tumorigenesis, embryonic stem cell differentiation, and viral infection (Kennedy et al., 2016). In mouse GC-1 SPG cells, meclofenamic acid inhibits spermatogonial proliferation by affecting CDKs expression through a m^6^A-dependent mRNA degradation pathway (Huang et al., 2019). In liver cancer, overexpression of METTL3 leads to the degradation of suppressor of cytokine signalling two and promotes tumor growth (Lin et al., 2019).

M^6^A modification is also associated with the pathophysiological processes of cardiac differentiation and a variety of cardiovascular diseases. Methyltransferase-like 3 (METTL3) is a major factor involved in abnormal m^6^A modification. Silencing METTL3 enhances the autophagic flux and represses apoptosis in hypoxia/reoxygenation-treated cardiomyocytes (Song et al., 2019). Overexpression METTL3 increased m^6^A levels in mRNAs isolated from transverse aortic constriction (TAC) mice hearts. TAC inducd pathological hypertrophic cellular growth was attenuated in hearts of METTL3-overexpressing mice, as evidenced by the crosssectional area of myocytes (Kmietczyk et al., 2019). In addition, aging caused METTL3/METTL14 down-regulation in aorta and atria in male animals while the differentiation-induced increased level of METTL16 (Arcidiacono et al., 2020). Prabhu M et al. found that downregulation of fat mass and obesity associated protein (FTO) expression in failing mammalian hearts and hypoxic cardiomyocytes increased the m^6^A modification of RNAs and reduced the contractile function of cardiomyocytes (Mathiyalagan et al., 2019). FTO also played a vital role in cardiac contractile function during homeostasis and remodeling. FTO overexpression attenuated the ischemia-induced elevation in m^6^A modification and significantly improved cardiac function of post-myocardial infraction (Mathiyalagan et al., 2019). Berulava et al. reported that differently expressed genes of m^6^A methylation are involved in heart failure development. Bioinformatics analysis has revealed that the differentially m^6^A are mainly involved in metabolism and cardiac signaling (Berulava et al., 2020). However, the regulatory role of the m^6^A modification in cardiac function is still unclear.

The contraction of the ventricle transports blood to the capillaries of the body or lungs, while the contraction of the atria send blood into the ventricle. In addition, the right ventricle mainly supplies blood to the lungs, and the blood in the right ventricle is venous blood. The left ventricle mainly supplies blood to the organs and tissues of the body, and the blood in the left ventricle is arterial blood. Therefore, the function of the left ventricle is very important to maintain organ perfusion. If the left ventricular function declines, it will leads to organ perfusion insufficiency and, eventually, organ failure. In this direction, we focused on the mechanism underlying left ventricle injury in sepsis to find any potential clinical significance for improving organ perfusion. The overall goal of this study was to explore a new layer of epigenetic alterations through the genome-wide screening of the altered m^6^A-tagged transcript profiles in lipopolysaccharide (LPS)-induced myocardial dysfunction. We successfully mapped m^6^A transcripts in left ventricle tissue in LPS-induced sepsis. Some potential roles of the m^6^A modification in the physiological and pathological mechanisms of sepsis were revealed.

## Materials and Methods

### Animals

Eight-week-old male Wistar rats weighing 250–350 g were used in this study. All the rats used in our study were obtained from Charles River Laboratories (Beijing, China). Water and standard laboratory food were freely available to the animals. The Experimental Animal Welfare Ethics Branch and the Biomedical Ethics Committee of Peking University approved our study protocol (LA 2020343). All the procedures for handling the animals were in accordance with the Guide for the Care and Use of Laboratory Animals (NIH Publication No. 85–23, revised 1996).

After acclimating for 1 week, 10 Wistar rats were randomly divided into two groups. The rats in the LPS group (*n* = 5) were intraperitoneally injected with LPS (10 mg/kg, 5 mg LPS dissolved in 1 ml 0.9% saline). The rats in the control group (*n* = 5) were intraperitoneally injected with 0.9% saline (2 ml/kg). 24 hours after LPS injection, mean blood pressure was measured by the tail-cuff method with a noninvasive blood pressure measurement system. The left ventricle tissue samples were immediately transferred to liquid nitrogen and stored at −80°C for preservation.

### RNA Extraction and Quality Control

Total RNA was isolated from the left ventricle tissues of the LPS (*n* = 5) and control (*n* = 5) groups using TRIzol Reagent (Invitrogen, United States). The quantity and purity of the total RNA samples were measured by a NanoDrop ND-1000 (ThermoFisher, United States).

### Quantification of Global m^6^A Levels

Liquid chromatography-tandem mass spectrometry (LC-MS/MS)-based mRNA modification detection was performed according to the Aksomics standard protocol. Briefly, 5 μg of total RNA from the heart tissues of the LPS (*n* = 5) and control (*n* = 5) groups was used to isolate mRNA using the NEBNext Poly(A) mRNA Magnetic Isolation Module (NEB, E7490, United States). The purified mRNA was quantified using the Qubit RNA HS Assay kit (ThermoFisher, United States) and digested to single dephosphorylated nucleosides by an enzyme mixture. Pretreated nucleosides solution was deproteinized using Satorius 10,000-Da MWCO spin filter. LC-MS/MS analysis was performed on an Agilent 6460 QQQ mass spectrometer with an Agilent 1260 HPLC system using Multi reaction monitoring (MRM) detection mode (Agilent, United States. The nucleosides were quantified by using retention time and the nucleoside to base ion mass transitions of 268-136 (A) and 282-150 (m^6^A). Quantification was performed in comparison with the standard curve obtained from pure nucleoside standards running with the same batch of samples. The m^6^A level was calculated as the ratio of m^6^A to A based on the calibrated concentrations.

### Quantitative Real-Time PCR

After extraction, the total RNA was reverse-transcribed into cDNA using SuperScriptTM III Reverse Transcriptase (Invitrogen) according to the manufacturer’s instructions. Amplification and detection were performed using 2× SYBR Green PCR Master Mix (Arraysta, United States) on a ViiA 7 Real-Time PCR System (Applied Biosystems). The sequences of the gene-specific primers are listed in Supplementary Table S1. GAPDH was used as the control housekeeping gene.

### Western Blot Analysis

Protein extraction of left ventricle tissue and the protein concentration was determined by Bradford method (M&C Gene Technology Ltd.). The protein samples were then subjected to SDS-polyacrylamide gel electrophoresis (SDS-PAGE) and transferred to polyvinylidene difluoride membrane. The membranes were probed with antibodies of interest. The antibodies used were as follows: METTL3, METTL14, Wilms tumor 1-associated protein (WTAP), and YTH N6-methyladenosine RNA binding protein 1 (YTHDF1), YTHDF3 (all1:1,000; Cell Signal Technology), and β-actin (1:4,000; Abcam).

### M^6^A mRNA&lncRNA Epitranscriptomic Microarray

Sample preparation and microarray hybridization were performed based on Arraystar’s standard protocols (Arraystar). Briefly, total RNA from the heart tissues of the LPS (n = 5) and control (*n* = 5) groups was immunoprecipitated with an anti-m^6^A antibody (Synaptic Systems, 202003). The modified RNAs immunoprecipitated by the magnetic beads were labelled as “IP”, and the unmodified RNAs in the supernatant were labelled as “Sup”. The “IP” and “Sup” RNAs were labeled with Cy5 and Cy3, respectively, as cRNAs in separate reactions using the Arraystar Super RNA Labeling Kit (ArrayStar). The cRNAs were combined and hybridized onto an Arraystar Rat mRNA&lncRNA Epitranscriptomic Microarray (4 × 44K, Arraystar) that contained 27,770 mRNA and 10,582 lncRNA degenerate probes. The hybridized arrays were scanned in two-color channels by an Agilent Scanner G2505C.

### Microarray Data Analysis

Agilent Feature Extraction software (version 11.0.1.1, United States) was used to analyze the acquired array images. Each probe signal was evaluated and flagged as present, absent or marginal in at least 5 out of 10 samples. The raw intensities of the IP (immunoprecipitated, Cy5-labeled) and Sup (supernatant, Cy3-labeled) samples were normalized by the average of log2-scaled spike-in RNA intensities. The differentially m^6^A-methylated RNAs between the LPS and control groups were identified by filtering with thresholds of fold change >1.5 and statistical significance (*p* < 0.05).

### Bioinformatics Analysis

Gene Ontology (GO) and Kyoto Encyclopedia of Genes and Genomes (KEGG) analyses were performed using some package in the R environment for statistical computing and graphics. The differentially m^6^A-methylated mRNAs, as well as the downstream mRNAs predicted by the competing endogenous RNA (ceRNA) network, were classified into different GO terms and enriched in certain biological pathways. Protein-protein interaction (PPI) analysis was performed using the STRING database (https://string-db.org). The SRAMP database (http://www.cuilab.cn/sramp) was used to predict the number of m^6^A sites on the differentially m^6^A-methylated transcripts (Zhou et al., 2016).

### M^6^A Single-Base Site qPCR

Before amplification and detection, the LPS (*n* = 5) and control (*n* = 5) group samples were treated with an *Escherichia coli* toxin and RNA endoribonuclease, MazF. MazF is reported to be sensitive to m^6^A modification within the ACA motif (Imanishi et al., 2017). The MazF-digested mRNA samples and the nondigested samples were subjected to reverse transcription using SuperScriptTM III Reverse Transcriptase (Invitrogen) for qPCR as described above. The targeted lncRNAs and mRNAs were predicted by SRAMP to identify the ACA motif and m^6^A position. The primer sequences specific for the methylated lncRNAs and mRNAs are listed in Supplementary Table S2. The relative expression levels were calculated using the 2^−△△Ct^ method, and the tested genes were calibrated with MazF as follows:

$$\%MazF-=\left(2^{\wedge }-Ct_{MazF+}\right)/\left(2^{\wedge }-Ct_{MazF-}\right)\times 100\%$$

### Competing Endogenous RNA Network Construction

The targeted lncRNAs were verified by m^6^A single-base site qPCR to construct the ceRNA network. Only three steps were used for ceRNA network construction. First, the potential target microRNAs of lncRNAs were predicted with Aksomics’s homemade miRNA target prediction software based on TargetScan and miRanda (Enright et al., 2003; Pasquinelli, 2012). Second, the target genes of the miRNAs involved in the lncRNA-miRNA interaction network were predicted with the database described above. Finally, the lncRNA-microRNA-mRNA interaction networks were constructed by Cytoscape v2.8.3. The targeted mRNAs were also analyzed by GO and KEGG to completely understand the ceRNA effects.

### Statistical Analysis

The statistical significance of the GO and KEGG analysis of mRNAs enrichment were calculated by Fisher’s exact test *p* < 0.05 and -log10(*p*) transformed as the enrichment score as well as mRNAs predicted by the ceRNA network for GO and KEGG analysis. The significance of the differences in the expression and methylation levels between the LPS and control groups was evaluated with an unpaired two-sided *t*-test for LC-MS/MS, qRT-PCR, microarray analysis and m^6^A single-base site qPCR, and the recommended *p*-value threshold was less than 0.05. “*” indicates a significant difference compared with control group (*p* < 0.05).

## Results

### The Global Levels of m^6^A Modification Were Decreased

**FIGURE 1:**
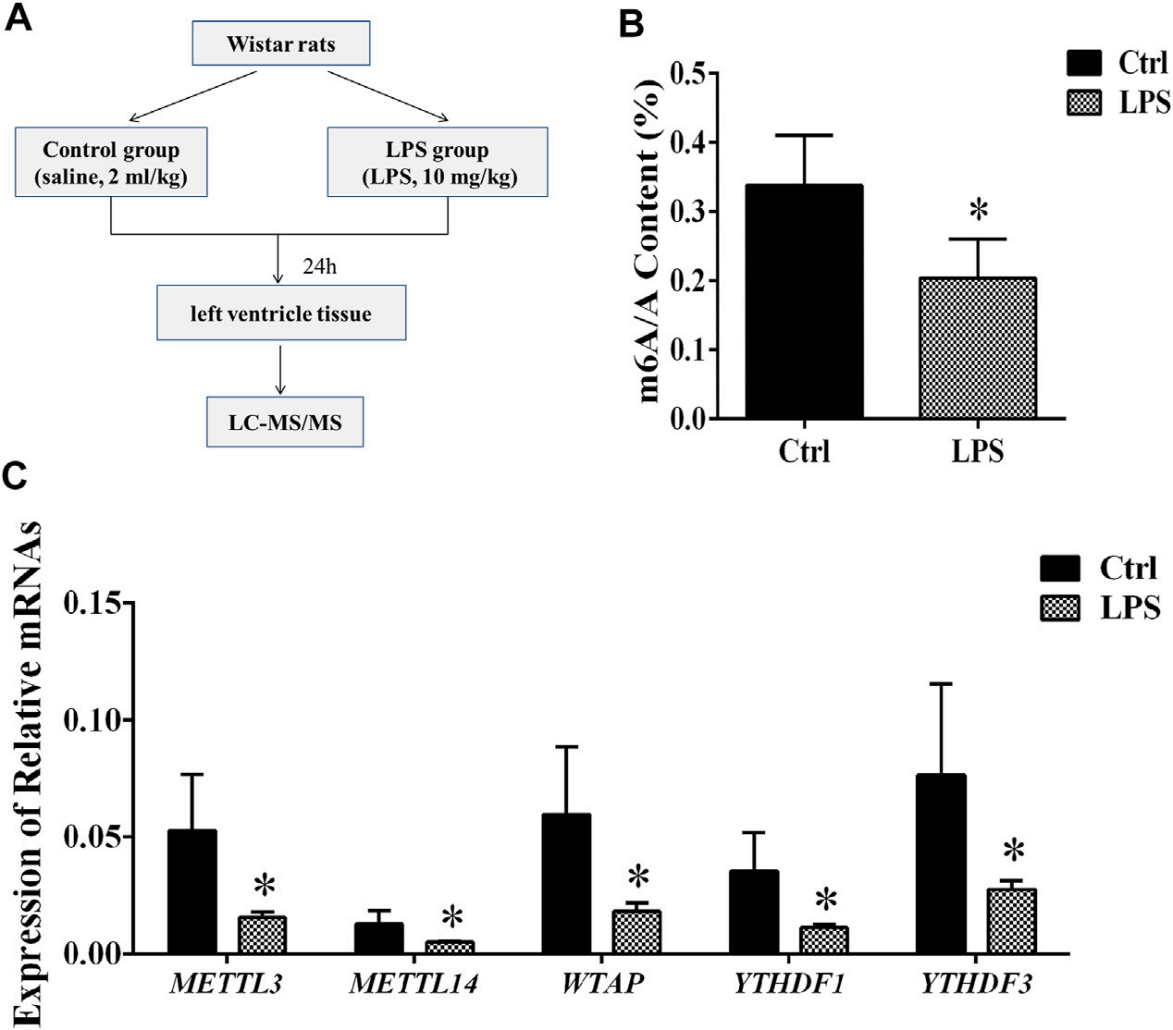
Global level of m^6^A modification and mRNA levels of enzymes in left ventricle tissue. **(A)** Flowchart of tissue collection. **(B)** Global level of m^6^A modification measured by LC-MS/MS in left ventricle tissue. **(C)** Relative expression mRNA levels of m^6^A-related enzymes in left ventricle tissue during sepsis. The mRNA levels were determined by qRT-PCR and normalized by GAPDH. *p < 0.05 versus the Ctrl group. Ctrl, control; LPS, lipopolysaccharide.

In about 24 h after giving intraperitoneal injection of LPS, we measured the MAP of rats. As previous paper, the rats with a MAP declined to 25–30% or below were chosen as the sepsis model (Nie et al., 2020). Furthermore, signs of shock such as lassitude, tachycardia and a sharp drop in body temperature were observed in all sepsis rats. In the control group, there was no significant change in MAP in rats injected with saline. Previous study showed that there is a positive relationship between MAP and heart function (Yang et al., 2018; Zhang et al., 2019). Therefore, we isolated heart tissue and chose the left ventricle for identification of m^6^A modification levels (Figure 1A). There was no measurement of cardiac function or sepsis physiology in these experiments.

To determine the difference in the m^6^A modification levels between the LPS and control groups, we used LC-MS/MS to measure these levels. We found that the global m^6^A modification level in the rat left ventricle tissues from the LPS group was significantly decreased compared with control group (Figure 1B).

### The Expression of m^6^A Writers and Readers Was Downregulated

The m^6^A methylation modification is dynamically modulated by RNA methyltransferases, RNA-binding proteins and demethylases. We analyzed the mRNAs level of RNA methyltransferases (writers: METTL3, METTL14, and WTAP), RNA-binding proteins (readers: YTHDF 1 and YTHDF 3) and a demethylase (eraser: FTO). It was found that the expression of the METTL3/METTL14/WTAP/YTHDF 1, and YTHDF 3 in the LPS group was significantly downregulated compared with control group. However, the expression of FTO has no significant difference (Figure 1C). In addition, we measured the protein expression of METTL3/14, WTAP, and YTHDF1/3. We found that protein expressions of these proteins significantly downregulated in LPS group (Supplementary Figure S1). The expressions of METTL3/14, WTAP, and YTHDF1/3 were decreased by 42.2, 64, 47.6, 49.6 and 56.6%, respectively. The results of protein expression and mRNA expression are consistent.

### M^6^A Modification Profiles of lncRNAs and mRNAs

**FIGURE 2:**
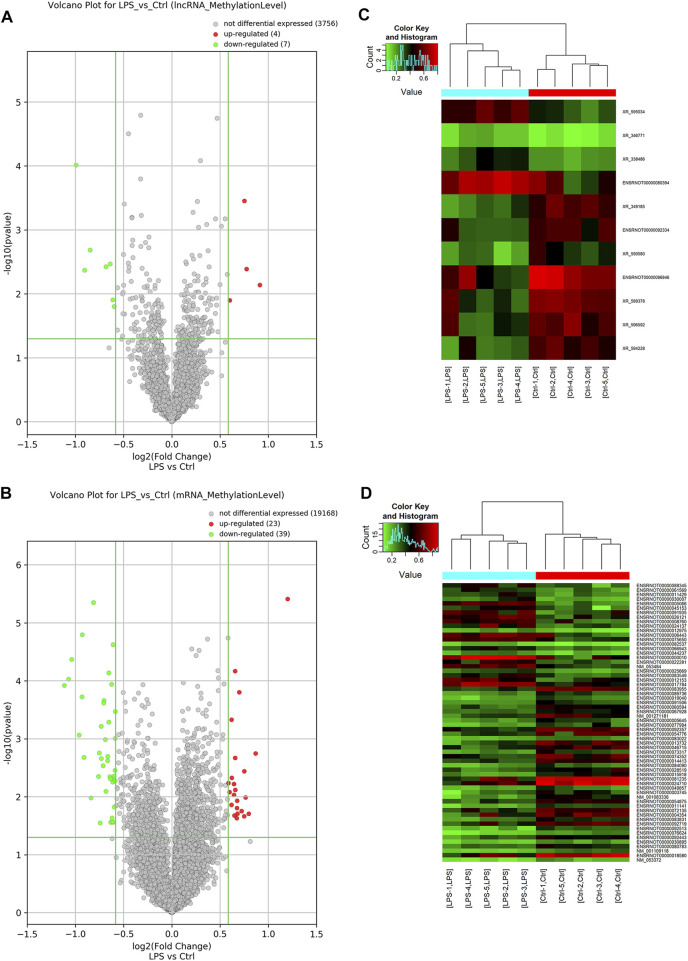
M^6^A modification profiles of lncRNA& mRNA in LPS and control group. **(A)** Volcano plots showing the lncRNAs that were differentially methylated between LPS and control group with statistical significance. **(B)** Volcano plots showing the mRNAs that were differentially methylated between LPS and control group with statistical significance (fold changes ≥1.5 and *p* < 0.05). **(C)** Hierarchical clustering analysis the differentially methylated lncRNAs. **(D)** Hierarchical clustering analysis the differentially methylated mRNAs. Ctrl, control; LPS, lipopolysaccharide.

Probes specific for 27,770 mRNAs and 10,582 lncRNAs were used to analyze the samples from the LPS and control groups using an m^6^A mRNA and lncRNA Arraystar epitranscriptomic microarray. The results showed that the m^6^A modification levels of 62 mRNAs and 11 lncRNAs were significantly altered in the LPS group compared with control group (fold change >1.5, *p*-value < 0.05) (Figures 2A,B). The altered lncRNAs and mRNAs were aligned for cluster analysis (Figures 2C,D). Based on their fold changes, we selected 11 lncRNAs with significantly altered m^6^A modification levels and the top 10 up- and downregulated mRNAs to present the results (Tables 1, 2).

**TABLE 1:** The detailed information of the hyper-methylated and hypo-methylated lncRNAs.

Transcript_ID	Type	Gene symbol	RNA length	Locus	Regulation	Fold change	*p*-value
XR_346771	lncRNA	LOC102552786	1764	chr5:148464894–148467759:−	Hyper	1.882651632	0.007280323
XR_338486	lncRNA	LOC102547952	997	chr10:14824098–14825718:+	Hyper	1.711575396	0.004096892
XR_595034	lncRNA	LOC103693543	1931	chr15:32741179–32746755:−	Hyper	1.684697484	0.000352,285
ENSRNOT00000080394	lncRNA	AABR07059875.4	185	chr4:39636928–39637206:−	Hyper	1.516656629	0.012635768
XR_349185	lncRNA	LOC102548542	1841	chr9:93297368–93301959:−	Hypo	0.502693743	0.000096980
XR_590580	lncRNA	LOC103691283	540	chr12:22434074–22434701:−	Hypo	0.534479482	0.004268491
XR_594228	lncRNA	LOC103690476	702	chr8:130823601–130827254:+	Hypo	0.555894587	0.00206,689
XR_589378	lncRNA	LOC103690602	719	chr11:79090944–79093774:+	Hypo	0.623167691	0.00376,447
ENSRNOT00000092334	lncRNA	Comp	991	chr16:20803585–20805482:−	Hypo	0.642164861	0.003406754
ENSRNOT00000086846	lncRNA	AABR07026021.1	524	chr16:59517000–59524544:+	Hypo	0.654135708	0.012402534
XR_596592	lncRNA	LOC102548402	5498	chr17:5486419–5494314:−	Hypo	0.660712559	0.015797815

**TABLE 2:** The detailed information of the top ten hyper-methylated and top ten hypo-methylated mRNAs.

Transcript_ID	Type	GeneSymbol	RNA length	Locus	Regulation	Fold change	*p*-value
ENSRNOT00000005066	protein_coding	Cfap52	2237	chr10:54470835–54512169:−	Hyper	2.299100294	0.000003881
ENSRNOT00000000010	protein_coding	Tmco5b	1361	chr3:104749051–104765436:+	Hyper	1.8263071	0.001794981
ENSRNOT00000030007	protein_coding	Ptk2b	3848	chr15:42827310–42947656:−	Hyper	1.738045027	0.019677042
ENSRNOT00000006443	protein_coding	Rtn4	4665	chr14:114126966–114174458:+	Hyper	1.699559099	0.010322126
ENSRNOT00000008760	protein_coding	Cd44	1437	chr3:92697833–92749121:−	Hyper	1.683312914	0.003639311
ENSRNOT00000045153	protein_coding	LOC103689983	1223	chrX:158623240–158655198:	Hyper	1.68214218	0.021749985
ENSRNOT00000082537	protein_coding	Clec1b	958	chr4:163162211–163170466:+	Hyper	1.651427112	0.017707274
NM_053484	protein_coding	Gas7	6796	chr10:54086843–54240798:+	Hyper	1.623629355	0.000157904
ENSRNOT00000024137	protein_coding	Drd4	1416	chr1:214278296–214281483:+	Hyper	1.603101886	0.019629213
ENSRNOT00000088345	protein_coding	AABR07052508.1	258	chr3:58380143–58386375:+	Hyper	1.599183502	0.011739780
NM_001083336	protein_coding	Stk38l	2406	chr4:181027212–181087530:+	Hypo	0.461936557	0.000120507
ENSRNOT00000076624	protein_coding	Slpil3	663	chr3:160774855–160777092:−	Hypo	0.476197276	0.000093915
ENSRNOT00000015918	protein_coding	Tnfrsf21	4350	chr9:20546159–20621051:	Hypo	0.487217803	0.000042853
ENSRNOT00000092513	protein_coding	Rps15a	528	chr1:187759865–187766670:	Hypo	0.513673869	0.000863271
ENSRNOT00000011141	protein_coding	Gprc5d	1402	chr4:168872897–168884886:−	Hypo	0.524397823	0.000189455
NM_001109118	protein_coding	Elovl2	3731	chr17:21382461–21422410:+	Hypo	0.525563958	0.000016059
ENSRNOT00000013732	protein_coding	Il6	1045	chr4:3043231–3047807:+	Hypo	0.532128419	0.002095281
ENSRNOT00000077994	protein_coding	Ssr1	1196	chr17:27496353–27510918:+	Hypo	0.559623758	0.010488981
ENSRNOT00000083955	protein_coding	Fkbp11	717	chr7:140398253–140401686:−	Hypo	0.569855508	0.000004472
NM_053372	protein_coding	Slpi	667	chr3:160799979–160802228:−	Hypo	0.590419522	0.004477119

The data that support the findings of this study are openly available in the GenBank databases under accession number GSE159309.

### GO and KEGG Analyses of Differentially Methylated mRNAs

**FIGURE 3:**
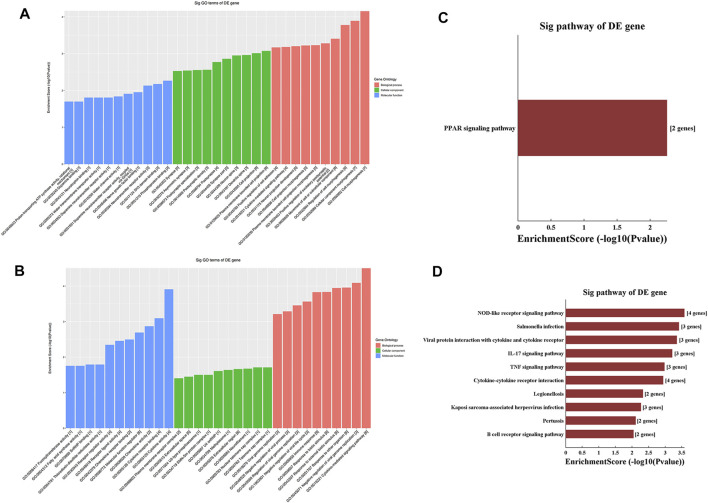
Gene ontology enrichment and Kyoto Encyclopedia of Genes and Genomes analysis of altered m^6^A transcripts. **(A)** The top ten significantly enriched GO terms of upregulation m^6^A transcripts. **(B)** The top ten significantly enriched GO terms of downregulation m^6^A transcripts. **(C)** The significantly enriched KEGG pathways of upregulation m^6^A transcripts. **(D)** The significantly enriched KEGG pathways of downregulation m^6^A transcripts. GO, Gene ontology; KEGG, Kyoto Encyclopedia of Genes and Genomes. DE, differentially expressed.

GO analysis of the mRNAs with increased and decreased m^6^A modification levels was performed. Among the mRNAs with decreased m^6^A modification levels, the “cytokine-mediated signaling pathway” in BP, “immune response” in BP and “cytokine activity” in MF were revealed to have the maximum enrichment scores for GO terms (Figures 3A,B; Tables 3, 4).

**TABLE 3:** Gene Ontology analysis of the top ten hyper-methylated mRNAs.

GO.ID	Term	Ontology	Count	*p*-value	Fdr	Enrichment score
GO:0000902	Cell morphogenesis	Biological process	7	7.0344E-05	0.324473551	4.152772946
GO:0032989	Cellular component morphogenesis	Biological process	7	0.000128477	0.324473551	3.891174337
GO:0022604	Regulation of cell morphogenesis	Biological process	5	0.000166488	0.324473551	3.778618248
GO:0006928	Movement of cell or subcellular component	Biological process	8	0.000393	0.324473551	3.405607496
GO:2000463	Positive regulation of excitatory postsynaptic potential	Biological process	2	0.000525054	0.324473551	3.279795677
GO:0120,039	Plasma membrane bounded cell projection morphogenesis	Biological process	5	0.00058802	0.324473551	3.230607569
GO:0048858	Cell projection morphogenesis	Biological process	5	0.000599981	0.324473551	3.22186279
GO:0031175	Neuron projection development	Biological process	6	0.000627138	0.324473551	3.202636833
GO:0019221	Cytokine-mediated signaling pathway	Biological process	4	0.000657648	0.324473551	3.18200643
GO:0045785	Positive regulation of cell adhesion	Biological process	4	0.00067698	0.324473551	3.169424277
GO:0120,025	Plasma membrane bounded cell projection	Cellular component	8	0.000840205	0.232167614	3.075614779
GO:0042995	Cell projection	Cellular component	8	0.000972547	0.232167614	3.012089301
GO:0043197	Dendritic spine	Cellular component	3	0.001088661	0.232167614	2.963107415
GO:0044309	Neuron spine	Cellular component	3	0.001120031	0.232167614	2.950770139
GO:0044456	Synapse part	Cellular component	5	0.001379925	0.232167614	2.860144529
GO:0098794	Postsynapse	Cellular component	4	0.001689647	0.232167614	2.772204087
GO:0014069	Postsynaptic density	Cellular component	3	0.002740157	0.232167614	2.562224576
GO:0099572	Postsynaptic specialization	Cellular component	3	0.002796098	0.232167614	2.55344754
GO:0032279	Asymmetric synapse	Cellular component	3	0.002881334	0.232167614	2.540406329
GO:0045202	Synapse	Cellular component	5	0.002960384	0.232167614	2.528652018
GO:0051219	Phosphoprotein binding	Molecular function	2	0.005431961	1	2.265043371
GO:0017124	SH3 domain binding	Molecular function	2	0.006663867	1	2.176273674
GO:0030594	Neurotransmitter receptor activity	Molecular function	2	0.007384559	1	2.131675429
GO:0048406	Nerve growth factor binding	Molecular function	1	0.011176355	1	1.951699809
GO:0001591	Dopamine neurotransmitter receptor activity, coupled via Gi/Go	Molecular function	1	0.012287459	1	1.910537933
GO:0015250	Water channel activity	Molecular function	1	0.014506118	1	1.838448788
GO:0004952	Dopamine neurotransmitter receptor activity	Molecular function	1	0.015613677	1	1.806494817
GO:0005372	Water transmembrane transporter activity	Molecular function	1	0.015613677	1	1.806494817
GO:0043121	Neurotrophin binding	Molecular function	1	0.015613677	1	1.806494817
GO:0035240	Dopamine binding	Molecular function	1	0.02003213	1	1.698272881

**TABLE 4:** Gene Ontology analysis of the top ten hypo-methylated mRNAs.

GO.ID	Term	Ontology	Count	*p*-value	Fdr	Enrichment score
GO:0019221	Cytokine-mediated signaling pathway	Biological process	6	3.15545E-05	0.16723586	4.500939235
GO:0045071	Negative regulation of viral genome replication	Biological process	3	8.13812E-05	0.16723586	4.089475756
GO:0051707	Response to other organism	Biological process	8	0.000110304	0.16723586	3.957409854
GO:0043207	Response to external biotic stimulus	Biological process	8	0.000113542	0.16723586	3.944843243
GO:0009607	Response to biotic stimulus	Biological process	8	0.000146422	0.16723586	3.834392194
GO:0006955	Immune response	Biological process	9	0.000147975	0.16723586	3.829813087
GO:1903901	Negative regulation of viral life cycle	Biological process	3	0.000272286	0.263767544	3.564974349
GO:0045069	Regulation of viral genome replication	Biological process	3	0.000350815	0.297359886	3.454921375
GO:0048525	Negative regulation of viral process	Biological process	3	0.000516858	0.389424083	3.286628429
GO:0019079	Viral genome replication	Biological process	3	0.000615908	0.39115453	3.210484097
GO:0000782	Telomere cap complex	Cellular component	1	0.019429856	1	1.711530414
GO:0000783	Nuclear telomere cap complex	Cellular component	1	0.019429856	1	1.711530414
GO:0005883	Neurofilament	Cellular component	1	0.021177942	1	1.674116247
GO:0005576	Extracellular region	Cellular component	8	0.021620359	1	1.665137091
GO:0034709	Methylosome	Cellular component	1	0.022923006	1	1.63972844
GO:0005687	U4 snRNP	Cellular component	1	0.024665052	1	1.607917962
GO:0034719	SMN-Sm protein complex	Cellular component	1	0.031603169	1	1.500269361
GO:0071004	U2-type prespliceosome	Cellular component	1	0.031603169	1	1.500269361
GO:0005615	Extracellular space	Cellular component	6	0.035593759	1	1.448626141
GO:0098802	Plasma membrane receptor complex	Cellular component	2	0.039506187	1	1.40333488
GO:0005125	Cytokine activity	Molecular function	4	0.000123343	0.1554123	3.908885157
GO:0005126	Cytokine receptor binding	Molecular function	4	0.000804967	0.507128913	3.094222177
GO:0008009	Chemokine activity	Molecular function	2	0.001352491	0.568046338	2.868865526
GO:0098772	Molecular function regulator	Molecular function	8	0.002030662	0.639658597	2.692362313
GO:0042379	Chemokine receptor binding	Molecular function	2	0.003183034	0.733701618	2.497158668
GO:0048018	Receptor ligand activity	Molecular function	4	0.003493817	0.733701618	2.456699818
GO:0030545	Receptor regulator activity	Molecular function	4	0.004505466	0.810983968	2.346260236
GO:0004791	Thioredoxin-disulfide reductase activity	Molecular function	1	0.016139095	1	1.792120819
GO:0070990	snRNP binding	Molecular function	1	0.016139095	1	1.792120819
GO:0004312	Fatty acid synthase activity	Molecular function	1	0.017593822	1	1.754639806

KEGG analysis of the mRNAs with increased and decreased m^6^A methylation modification levels were also conducted. The results showed that the mRNAs with increased m^6^A modification levels were enriched in only one pathway, namely, the “PPAR signaling pathway” (Figure 3C). The mRNAs with decreased m^6^A modification levels were enriched in 27 pathways, and the following main pathways reached higher enrichment scores: “IL-17 signaling pathway”, “TNF signaling pathway” and “Cytokine-cytokine receptor interaction” (Figure 3D).

### qPCR Results of a Single-Base m^6^A Site

**FIGURE 4:**
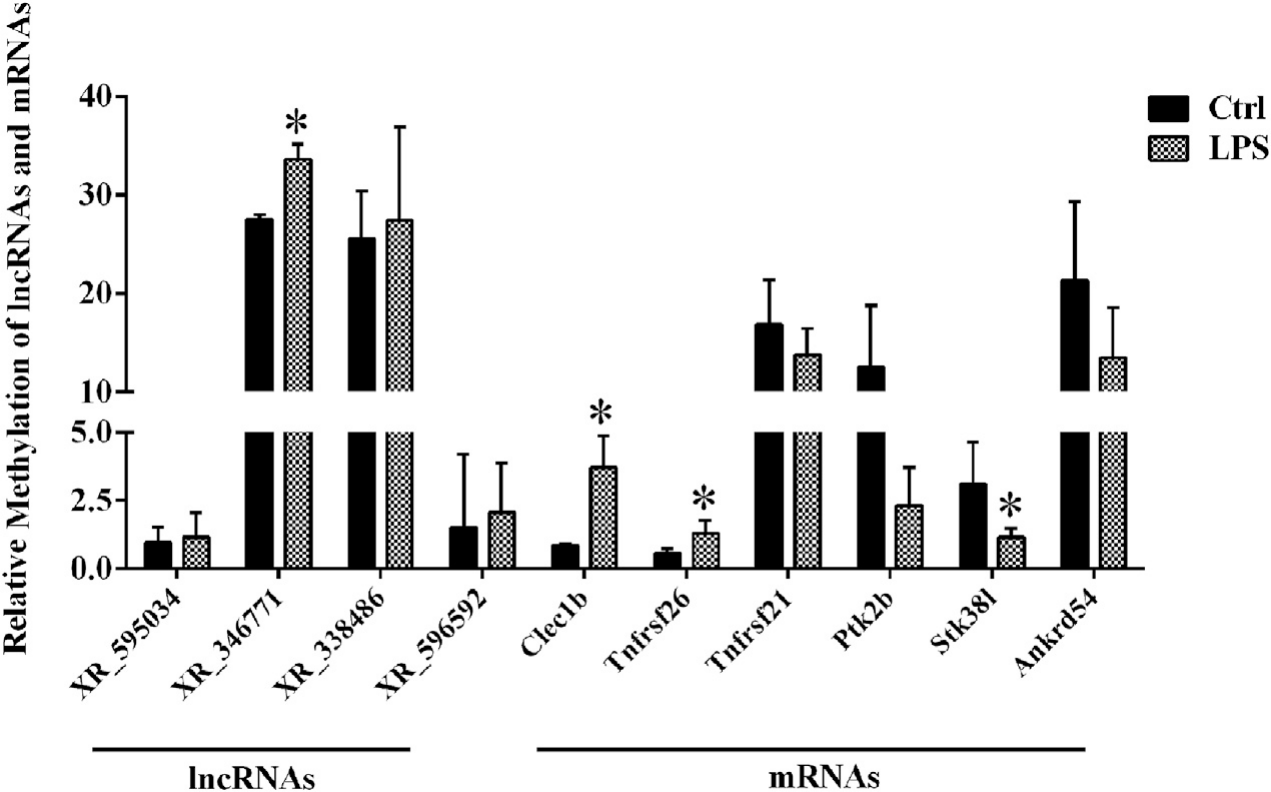
M^6^A single-base site qPCR was used to confirm the microarray data for the top four methylated lncRNAs and top six methylated mRNAs in left ventricle tissue between the LPS and control groups. *p < 0.05 versus the Ctrl group. Ctrl, control; LPS, lipopolysaccharide.

By using the SRAMP database, we predicted the mRNAs and lncRNAs with differential m^6^A modification in the order from high to low fold change of m^6^A modification. As a result, we identified four lncRNAs and six mRNAs with m^6^A ACA sites and with high confidence scores of qPCR validation of single-base m^6^A sites. The Detail information of lncRNAs and mRNAs detected by m^6^A single-base site qPCR was shown in Table 5. The results showed that among the lncRNAs, m^6^A modification levels were significantly increased in XR_346771. Among the mRNAs, m^6^A modification levels were significantly increased in C-type lectin domain family 1 member B (Clec1b) and tumor necrosis factor receptor superfamily member 26 (Tnfrsf26) and significantly decreased in serine/threonine kinase 38 like (STK38L) (Figure 4).

**TABLE 5:** Detail information of lncRNAs and mRNAs with hyper- and hypo-methylation detected by m^6^A single-base site qPCR.

GeneSymbol	Transcript_ID	Position	Sequence context	Score	Decision
LOC103693543	XR_595034	1359	GAGAG AUCUA CUGAA	0.674	m^6^A site (very high confidence)
			CAUCU GG**A**CA ACCUC		
			ACAUC AUACC AUCUC		
LOC102552786	XR_346771	1712	GGUCC UCCAA UGAAU	0.707	m^6^A site (very high confidence)
			UCAAA GG**A**CA AUAGG		
			CGACC ACCAG UGAAU		
LOC102547952	XR_338486	1546	CUGCA ACAUG GGAAG	0.676	m^6^A site (very high confidence)
			AAGAG GG**A**CA UAAGA		
			GAGGA CCCCA CCCCC		
LOC102548402	XR_596592	3965	AUCUG ACGGC AGGAU	0.677	m^6^A site (very high confidence)
			UUGGA GG**A**CA CCUCU		
			GAAAG GGCCC CAGAA		
Clec1b	ENSRNOT00000082537	831	ACCCA GCTTC CTGTA CAGAG AG**A**CA TTACT TAATA TGTGA GAGAA	0.731	m^6^A site (very high confidence)
Tnfrsf26	ENSRNOT00000066943	416	CAGGA ATGCA ATGCC ACAAT GG**A**CA CTGTG TGTGA CTCCA AGCAA	0.634	m^6^A site (high confidence)
Ptk2b	ENSRNOT00000030007	3278	GTGCT ACTTG GGCTA CATCT GG**A**CA GAAAG GACTC TGGGC ACAGA	0.732	m^6^A site (very high confidence)
Tnfrsf21	ENSRNOT00000015918	928	CTGGG GTGTG AGGAA GAAAG GG**A**CA GAGAA TGAAG ATGTG CGGTG	0.747	m^6^A site (very high confidence)
Stk38l	NM_001083336	2090	ACCGG CTGCA AGGAA CTTAA GG**A**CA CTGACTCCGA CATTA GAATT	0.640	m^6^A site (high confidence)
Ankrd54	ENSRNOT00000014413	1173	CCCTC TGGCT GTTAG GGAAG GG**A**CA GGAAC CCCAG AACAG AGGAA	0.643	m^6^A site (high confidence)

### PPI Analysis of Targeted Genes

**FIGURE 5:**
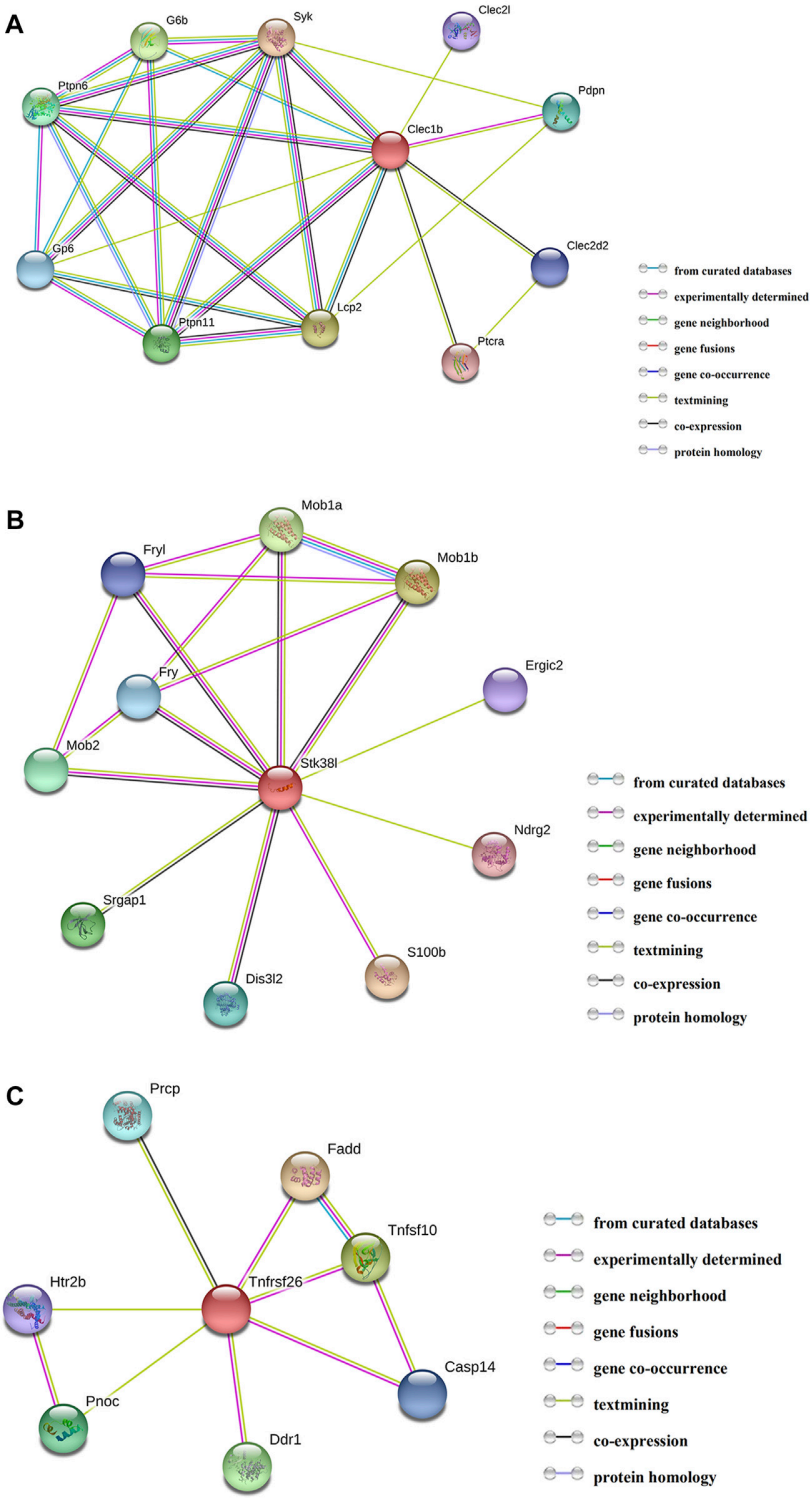
Protein–protein interaction analysis. **(A)** PPI network of Clec1b, **(B)** PPI network of Stk38l, **(C)** PPI network of Tnfrsf26. PPI, protein–protein interaction; Clec1b, C-type lectin domain family 1 member B; Tnfrsf26, tumor necrosis factor receptor superfamily member 26; STK38L, serine/threonine kinase 38 like.

By employing the STRING database, we performed PPI analysis of Clec1b, Stk38l, and Tnfrsf26 (Figures 5A–C). KEGG analysis revealed that the Clec1b interaction-related proteins were mainly enriched in “platelet activation” and other pathways. The Stk38l interaction-related proteins were mainly enriched in the “hippo signaling pathway”, and the Tnfrsf26 interaction-related proteins were mainly enriched in “apoptosis” (Table 6).

**TABLE 6:** Pathways of the matching proteins in the PPI network.

Target gene	Term ID	Term description	FDR	Matching proteins in the network
Clec1b	rno04650	Natural killer cell mediated cytotoxicity	0.000002500	Lcp2, Ptpn11, Ptpn6, Syk
	rno04611	Platelet activation	0.000360,000	Gp6, Lcp2, Syk
	rno04662	B Cell receptor signaling pathway	0.004900000	Ptpn6, Syk
	rno04664	Fc epsilon RI signaling pathway	0.004900000	Lcp2, Syk
	rno04660	T Cell receptor signaling pathway	0.006500000	Lcp2, Ptpn6
	rno04380	Osteoclast differentiation	0.007300000	Lcp2, Syk
	rno04072	Phospholipase D signaling pathway	0.009700000	Ptpn11, Syk
	rno04630	Jak-STAT signaling pathway	0.009700000	Ptpn11, Ptpn6
	rno05205	Proteoglycans in cancer	0.012500000	Ptpn11, Ptpn6
Stk38l	rno04392	Hippo signaling pathway - multiple species	0.000260000	Mob1a, Mob1b
	rno04390	Hippo signaling pathway	0.003500000	Mob1a, Mob1b
Tnfrsf26	rno04210	Apoptosis	0.023800000	Fadd, Tnfsf10
	rno04217	Necroptosis	0.023800000	Fadd, Tnfsf10

### ceRNA Analysis of lncRNA XR_346771

**FIGURE 6:**
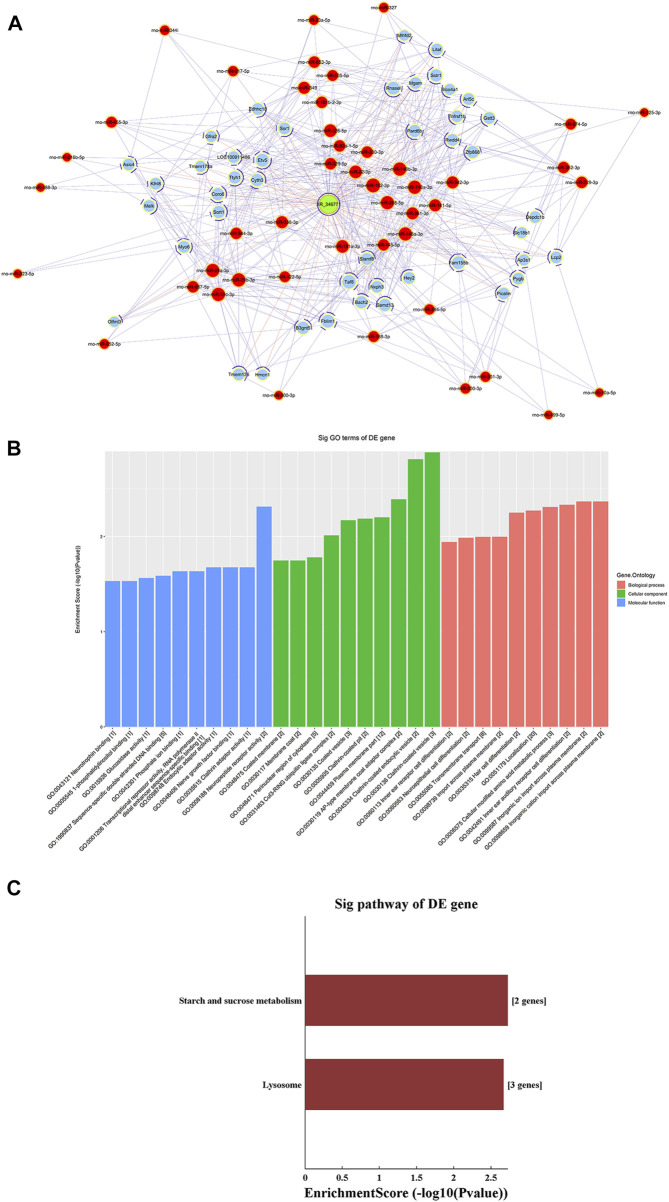
LncRNA XR_346,771-miRNA-mRNA ceRNA analysis. **(A)** The ceRNA network of XR_346,771–miRNA–mRNA. Red circles represent miRNAs, blue circles represent mRNAs, and green circles represent lncRNAs. **(B)** Ten most enriched GO terms involved in the ceRNA network. **(C)** KEGG pathways involved in the ceRNA network. ceRNA, competing endogenous RNA.

A ceRNA network of the lncRNA-microRNA-mRNA interaction network was constructed for XR_346,771 (Figure 6A). GO and KEGG analyses were performed on the predicted mRNAs (Figures 6B,C). The “inorganic cation import across plasma membrane”, “transmembrane transport”, and “import across plasma membrane” in BP were significantly enriched for GO terms (Figure 6B). FAM155B and PICALM are downstream proteins related to transmembrane transport that are regulated by XR_346771, and the corresponding sponged miRNAs are shown in Table 7.

**TABLE 7:** LncRNA XR_346771targeted miRNAs and mRNAs.

LncRNA	Targeted miRNA	Targeted mRNA
XR_346771	miR-146a-3p, miR-148a-3p	PICALM
	miR-148b-3p, miR-152-3p, miR-361-3p, miR-384-5p, miR-500-3p, miR-501-3p	
	miR-122-5p, miR-146a-3p, miR-188-3p, miR-298-5p, miR-326-5p, miR-329-5p, miR-361-3p, miR-500-3p, miR-501-3p, miR-542-3p, miR-674-5p, miR-92a-1-5p	FAM155B

## Discussion

Our study suggested that the m^6^A modification level was decreased in septic heart tissues. Our genome-wide screening of m^6^A-tagged transcript profiles indicated that the m^6^A modification levels in 39 mRNAs and 7 lncRNAs were significantly decreased and that the m^6^A modification levels in 23 mRNAs and four lncRNAs were significantly increased.

Pathway analyses of mRNAs with altered m6A modification were conducted in our study. The results showed that the mRNAs with decreased m^6^A modification levels were enriched in many pathways related to immune and inflammatory responses. In recent years, the roles of m^6^A modification in autoimmune and inflammatory diseases have attracted substantial attention (Rubio et al., 2018). METTL3 is a key enzyme of m^6^A methylation modification and is involved in immune and inflammatory regulation. Feng et al. found that METTL3 inhibits inflammation by affecting the alternative splicing of MyD88 (Feng et al., 2018). METTL3 expression is significantly upregulated in blood samples from patients with rheumatoid arthritis. In addition, METTL3 attenuates LPS-induced macrophage-mediated inflammation through the NF-κB pathway (Wang et al., 2019a). The m^6^A “reader” YT521-B homology domain family 2 (YTHDF2) inhibits HCC cells and tumor vasculature by degrading the mRNAs of IL11 and serpin family E member 2 (Wang et al., 2019b). These findings indicate the important role of m^6^A modification in inflammatory and immune disorders. Sepsis is mainly caused by the host’s unbalanced response to infection. In sepsis, microbial infections or necrotic tissues release a large amount of harmful substances, leading to the activation of the systemic immune response and the excessive activation of immune cells. The excessive release of cytokines is destructive. There is currently no effective treatment for sepsis because in the past few decades, attempts to use anti-inflammatory treatments to limit the tissue damage caused by excessive inflammation have failed (Zeni et al., 1997). Our study suggests that alterations in m^6^A modification in sepsis are closely related to inflammatory and immune responses. Since we study cardiac tissue, we speculate that m^6^A modification plays an important role in sepsis-induced cardiac function dysfunction.

We selected four lncRNAs and six mRNAs for the qPCR validation of single-base m^6^A sites in our study. The results showed that among the lncRNAs, the m^6^A modification levels were significantly increased in XR_346771. Among the mRNAs, the m^6^A modification levels were significantly increased in Clec1b and Tnfrsf26 and significantly decreased in Stk38l. These results are consistent with those of high-throughput sequencing; therefore, we attempted to conduct an in-depth analysis of this lncRNA and the three mRNAs.

First, we performed PPI analysis of Clec1b, STK38L, and Tnfrsf26 to investigate the downstream possible regulatory genes and biological functions of these proteins. The Clec1b-interacting proteins are enriched in the pathway of “platelet activation”. C-type lectin-like receptor 2 (CLEC-2) is a protein encoded by the Clec1b gene. Platelets play a critical role in innate and adaptive immunity (Semple et al., 2011). Platelets can alleviate LPS-induced septic shock by regulating the ability of macrophages to engulf and kill bacteria (Xiang et al., 2013). In two mouse models of sepsis (intraperitoneal LPS injection and cecal ligation and puncture), platelet CLEC-2 reduced the severity of sepsis by controlling the migration of monocytes/macrophages to the infection site, the expression of inflammatory mediators, and damage to organs (Rayes et al., 2017). In our sepsis model, we found that the m^6^A modification level of Clec1b mRNA was significantly increased. Considering that the degree and pattern of m^6^A modification may affect the transport, splicing, storage, translation, stability, and decay of mRNAs, we speculated that the elevated level of Clec1b m^6^A modification affects the functions of Clec1b and associate with activation of platelets. The interacting proteins of Clec1b were glycoprotein VI (Gp6), lymphocyte cytosolic protein 2 (Lcp2), and spleen tyrosine kinase (Syk). These proteins might be used as target proteins for in-depth research.

Our study suggests that the m^6^A modification level of Tnfrsf26 mRNA is increased and of Stk38l mRNA is decreased. According to PPI network analysis, Stk38l-interacting proteins are enriched in the “Hippo signaling pathway” pathway. Hippo signaling pathway may promote cell differentiation and death and inhibit cell proliferation (Chang et al., 2019). Tnfrsf26-interacting proteins are mainly enriched in the pathway of apoptosis. Sepsis-induced cardiac dysfunction has been reported by many studies to be closely related to apoptosis. When LPS stimulates H9C2 cells (rat embryonic cardiomyoblasts), the overexpression of miR-146a suppresses apoptosis and helps attenuate myocardial depression (An et al., 2018). Chao et al. found that the overexpression of Tid1-S can enhance ER-α to activate p-PI3K/p-Akt, attenuating LPS-induced apoptosis in H9C2 cells (Chao et al., 2019). In recent years, as the understanding of m^6^A modification has gradually deepened, studies have found that m^6^A modification plays an important role in the process of apoptosis. In the human glioma cell line U251, lower level of m^6^A modification can reduce apoptosis and promote cell proliferation (Li et al., 2019). Moreover, METTL3 can regulate the expression of several important proteins that regulate the survival, apoptosis, invasion, and proliferation of lung cancer cells (Lin et al., 2016). We hypothesized that the m^6^A modification of Tnfrsf26 and Stk38l might be related to cardiomyocyte apoptosis.

LncRNAs are a class of noncoding RNAs that are more than 200 nucleotides in length. In recent years, m^6^A-modified lncRNAs have received extensive attention. M^6^A modification may control gene expression by regulating the translation efficiency and stability of lncRNAs (Coker et al., 2019). For example, the m^6^A-modified lncRNA MALAT1 is implicated in ischemia reperfusion injury-induced inflammation (Yang et al., 2020). LncRNAs contain miRNA-responsive elements, which function as ceRNAs, interact with miRNAs and indirectly regulate mRNAs. In the present study, we established and analyzed a potential ceRNA network of XR_346771 through bioinformatics analysis. We performed GO and KEGG analyses of the predicted mRNAs to further explore the function of XR_346771.

GO analysis showed that “inorganic cation import across plasma membrane”, “inorganic ion import across plasma membrane”, “transmembrane transport”, and “import across plasma membrane” were significantly enriched. Cardiac contraction requires Ca^2+^, so abnormal Ca^2+^ homeostasis may play a key role in the pathogenesis of common cardiovascular diseases. Studies have reported that m^6^A modification promotes neurite extension and neuronal differentiation by acting as a Ca^2+^ channel (Mukobata et al., 2002). In 6-OHDA-induced PC12 cells and the cerebral striatum of rats with Parkinson’s disease, decreased m^6^A modification induces the expression of N-methyl-d-aspartate receptor 1 and increases oxidative stress and Ca^2+^ influx, leading to apoptosis of dopaminergic neurons (Chen et al., 2019). These studies indicate that m^6^A modification is involved in Ca^2+^ transport. We speculated that the m^6^A modification of XR_346771 might associate with abnormal Ca^2+^ homeostasis in cardiac tissues.

It is interesting that even though the methyltransferase complex was downregulated upon sepsis induced myocardial dysfunction; there were still 27 transcripts that were significantly hypermethylated. Chokkalla et al. found that, compared with sham group, global m^6^A increased significantly at 12 and 24 h of reperfusion in transient middle cerebral artery occlusion in C57BL/6J mice. The FTO decreased significantly after stroke compared with sham. While 139 transcripts (122 mRNAs and 17 lncRNAs) were hypermethylated, 8 transcripts (5 mRNAs and 3 lncRNAs) were hypomethylated in the ischemic brain at 12 h reperfusion (Chokkalla et al., 2019). Wang et al. found that METTL3 was downregulated after traumatic brain injury in mice. In total, 922 m^6^A peaks were differentially expressed as determined by m^6^A-RIP-seq, with 370 upregulated and 552 downregulated methylated mRNA (Wang et al., 2019b). These studies are consistent with our research. Previous research indicated that the methylation modification of m^6^A has been proved to be reversible, as it modulated by RNA methyltransferases, RNA-binding proteins and demethylases. The m^6^A methyltransferase, also known as “Writers”, including METTL3/14, WTAP, KIAA1429 and RNA binding motifs protein 15/15B (RBM15/15B), E3 ubiquitin-protein ligase Hakai (HAKAI) and zinc finger CCCH-type containing 13 (ZC3H13). The RNA-binding proteins, also known as “readers”, including: YTHDF1/2/3, YTH domain-containing reader proteins 1/2 (YTHDC1/2), insulin-like growth factor 2 mRNA-binding proteins 1/2/3 (IGF2BP1/2/3), epithelium-specific ETS (ESE) transcription factors (ELF3), heterogeneous nuclear ribonucleoprotein A2B1 (hnRNPA2B1). The m^6^A demethylase, also known as “eraser”, including: FTO, α-ketoglutarate-dependent dioxygenase alkB homologue 5 (ALKHB5). In our research we tested mRNA level of some enzymes, but not all enzymes. Therefore, there may be other enzymes involved in m^6^A modification upon sepsis induced myocardial dysfunction. The m^6^A modification status of specific RNA was catalyzed by different enzymes. METTL3 positively regulates expression of MYD88, a critical upstream regulator of NF-κB signaling, by facilitating m^6^A methylation modification to MYD88-RNA in mesenchymal stem cells (Yu et al., 2020). However, reduced expression of FTO increased Nanog mRNA m^6^A methylation under TNF-α stimulation, decreased Nanog mRNA and protein expression, and significantly inhibited mesenchymal stem cells capacity for differentiation to sweat gland cells (Wang et al., 2020). In our research, these transcripts that have undergone m^6^A modification might have been modified by different enzymes.

In conclusion, our experiments revealed that the level of m^6^A modification is significantly decreased in septic cardiac tissue. Through the genome-wide profiling of m^6^A-tagged mRNAs and lncRNAs and subsequent bioinformatics analysis, we revealed some potential functions of transcripts with altered m^6^A modification.

## Data Availability

The datasets presented in this study can be found in online repositories. The names of the repository/repositories and accession number(s) can be found below: https://www.ncbi.nlm.nih.gov/genbank/, GSE159309.

## Supplementary Material

Supplementary Figure 1 Expressions of m^6^A-related enzymes were detected by western blot analysis. *n* = 3-4; **P* < 0.05. Ctrl, control; LPS, lipopolysaccharide.

Supplementary Table 1 Sequence of primers used for qRT-PCR analysis of mRNA levels.

Supplementary Table 2 Sequence of primers used for m^6^A single base site qPCR analysis of relative mRNA methylation levels.

## Abbreviations

BP, biological processes; CC, cellular components; ceRNA, Competing endogenous RNA; ELF3, epithelium-specific ETS (ESE) transcription factors; FTO, Fat mass and obesity-associated protein; GO, Gene Ontology; HAKAI, E3 ubiquitin-protein ligase Hakai; HCC, epatocellular carcinoma; hnRNPA2B1, heterogeneous nuclear ribonucleoprotein A2B1; IGF2BP1/2/3, insulin-like growth factor 2 mRNA-binding proteins 1/2/3; KEGG, Kyoto Encyclopedia of Genes and Genomes; LC-MS/MS, Liquid chromatography-tandem mass spectrometry; lncRNAs, long non-coding RNAs; LPS, lipopolysaccharide; m6A, N6-methyladenosine; METTL14, methyltransferase-like 14; METTL3, methyltransferase like 3; MF, molecular functions; PPI, Protein-protein interaction; RBM15/15B, RNA binding motifs protein 15/15B; WTAP, Wilms tumor 1-associated protein; YTHDC1/2, YTH domain-containing reader proteins 1/2; YTHDF 2, YTH domain family 2; ZC3H13, zinc finger CCCH-type containing 13.
