# Supplementary material for: Lipopolysaccharide Alters the m6A Epitranscriptomic Tagging of RNAs in Cardiac Tissue
Source: Front Mol Biosci. 2021 Jul 28;8:670160. doi: 10.3389/fmolb.2021.670160 (PMC8355517; doi:10.3389/fmolb.2021.670160)

Supplementary Material

1 Supplementary Tables

Supplementary Table S1. Sequence of primers used for qRT-PCR analysis of mRNA levels

Gene Name	Sequence	Tm (°C)	Product Size (bp)
GAPDH	F:5’ GCTCTCTGCTCCTCCCTGTTCTA3'<br>R:5’ TGGTAACCAGGCGTCCGATA3’	60	124
METTL3	F:5’ TTGACTACAGTGGCTACCTTT3’<br>R:5’ CCTTGGCTGTTGTGGTATT3’	60	220
METTL14	F:5’ GAGTATGTTTGCGAAAGTGGG3’<br>R:5’ TTGTCTTTCCAGGATTGTTCTT 3’	60	84
WTAP	F:5’ GAAAAACTAAAGCAGCAACAG3’<br>R:5’ CGTAAACTTCCAGGCACTC3’	60	267
YTHDF1	F:5’ GCCAGGAGGAAGAGGAGGTA 3’<br>R:5’ AGACAGCACCAAGCATACAGC 3’	60	131
YTHDF3	F:5’ GCCATGCGAAGGGAGAGAA3’<br>R:5’ AGCTTCAGGACACAAAGTGCT3’	60	278
FTO	F:5’ GAGCGGGAAGCTAAGAAA 3’<br>R:5’ GCTGCCACTGCTGATAGAA 3’	60	100

METTL3, methyltransferase-like 3; METTL14, methyltransferase-like 14; WTAP, Wilms tumor 1-associated protein; YTHDF1, YTH domain family 1; YTHDF3, YTH domain family 3; FTO, fat mass and obesity associated protein.

Supplementary Table S2. Sequence of primers used for m^{6}A single base site qPCR analysis of relative mRNA methylation levels

Gene Name	Sequence	Tm (℃)	Product Size (bp)
XR_595034	F:5' ATCTCATCCTGCCGCTCCTT3'<br>R:5' TCTTCCGCCTCCAGCACTTA3'	60	276
XR_346771	F:5' GAATTTGCTGAGTAAACACCCTAT3'<br>R:5' CACTGGTGGTCGCCTATTGT3'	60	147
XR_338486	F:5' TGTGGAGGGATCTGGTTCTTG3'<br>R:5' CTGCGGGACTGTATGGAGTG3'	60	191
XR_596592	F:5' ATCTGACGGCAGGATTTGGAG 3'<br>R:5' TTGCACTTTGAGCGTGGACAT3'	60	85
Clec1b	F:5' GATTGGATTATCACGCCAGAA3'<br>R:5' ACTCTTGTCAGGGCAGCATT3'	60	201
Tnfrsf21	F:5' TGCCTTGACTGACCGAGAAT3'<br>R:5' CACTGCTTACACCGCACATC3'	60	141
Ptk2b	F:5' CCTGCCTTGCCTTTGGTTAT3'<br>R:5' GTGTCTGTGCCCAGAGTCCTT3'	60	147
Tnfrsf26	F:5' GCTGTCCTGAGGGCAGAGTC3'<br>R:5' TGACGGGAGTAGCGAATGAA3'	60	184
Ankrd54	F:5' TGGAGACCAAGAGCGAGCAG 3'<br>R:5' GAGCCGGATTTCCCAATTTC3'	60	226
Stk38l	F:5' ATTTGCCCTGTTGGTTTGGA3'<br>R:5' ATGTTGCGGTGGAGGTGAGA3'	60	182

2 Supplementary Figure

Supplementary Figure S1. Expressions of m^{6}A-related enzymes were detected by western blot analysis. n = 3-4; *P < 0.05. Ctrl, control; LPS, lipopolysaccharide.

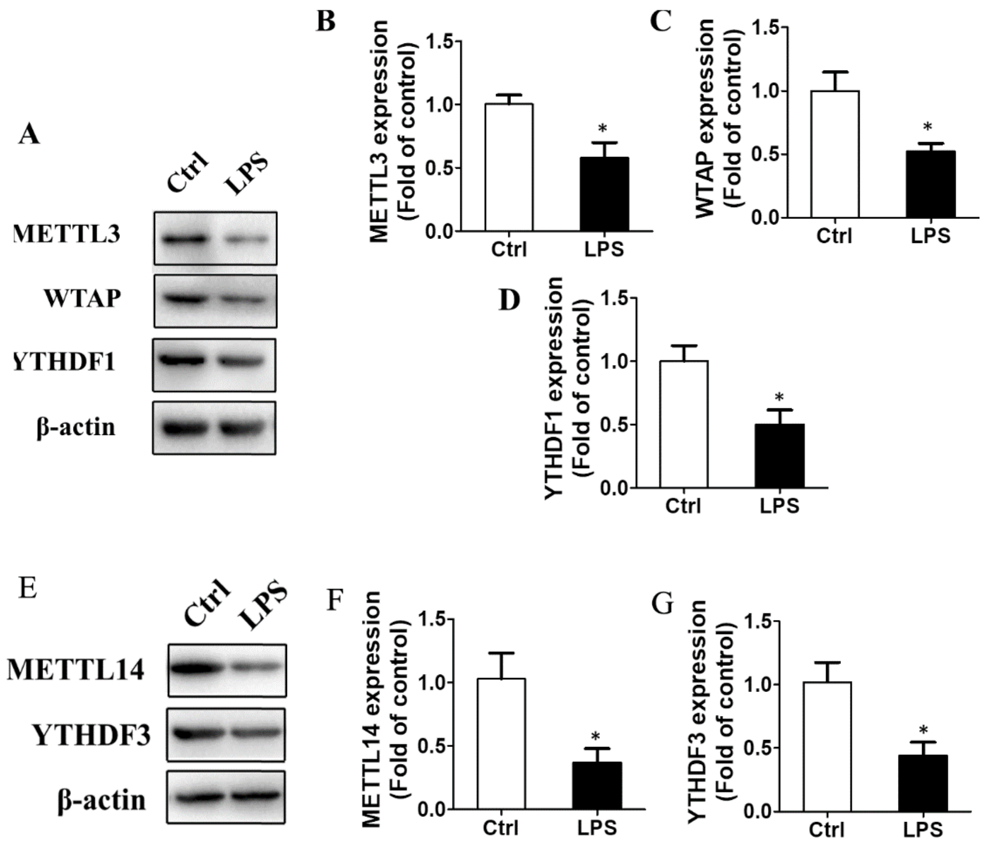

Supplement: Supplementary file 1 [file DataSheet1.pdf]
